# Music therapy and depression : the alternative approach

**DOI:** 10.1192/j.eurpsy.2023.900

**Published:** 2023-07-19

**Authors:** M. Zbidi, W. Bouali, M. Hnia, M. Kacem, R. Bensoussia, L. Zarrouk

**Affiliations:** 1 Département de psychiatrie, hôpital universitaire taher sfar de mahdia tunisie; 2 Psychiatry department, Taher sfar University hospital of mahdia tunisia, Mahdia, Tunisia

## Abstract

**Introduction:**

Depression is a highly prevalent disorder associated with reduced social functioning, impaired quality of life, and increased mortality. This disease is one of the most common reasons for the use of complementary and alternative therapies. Music therapy is a therapeutic approach that uses musical interaction as a means of communication and expression.

**Objectives:**

To assess, through a systematic review, the effectiveness of music therapy in patients with depression disorder, and to design a research protocol for a randomised controlled trial of group music therapy for depressed patients in a Psychiatry Department.

**Methods:**

We conducted a systematic review based on the Preferred Reporting Items for Systematic Reviews and Meta-analysis (PRISMA).We systematically searched 3 databases (Pubmed, Google Scholar and SciElo) and reviewed randomized controlled trials. The evaluation of the trials was made using the CONsolidated Standards of Reporting Trials (CONSORT) guidelines.The review included studies of 16-to-80-year-old impatients and outpatients of both genders with clinical depression using any diagnostic criteria such as ICD 10 (WHO 1992) or DSM 5 Research Diagnostic Criteria. Change in depressive symptoms was measured with various scales. An experimental protocol was then designed to conduct a randomized controlled trial for depressed patients in the Psychiatry Department at the University Hospital of Mahdia, seeking to supplement scientific knowledge in the field of music therapy that has not yet been explored.

**Results:**

A total of 13 articles were included in the study: The analysis of these articles highlighted a predominance of Anglo-Saxon papers and an increasing rate of publication over time.The duration of treatment varied between 2 weeks and 10 weeks and the number of music therapy sessions varied between 4 sessions and 20 sessions.Two major music therapy approach were identified, active method where patients are the ones making music and receptive or passive methods where patients will receive the music.12 researches included in our review reached the conclusion that music therapy had a significant positive effect on patients as the score scales were significantly lower after the end of the therapy. Only one included research found no significant difference between music therapy group and treatment as usual.In addition the results of all studies came on the conclusion that music therapy improved symptoms of anxiety and scores were significantly lower.

**Conclusions:**

This systematic review and meta-analysis suggests that music therapy has an effect on reducing depressive symptoms to some extent. However, high-quality trials evaluating the effects of music therapy on depression are required. Thus,the aim of our study protocol is to contribute to the development of this therapy

**Disclosure of Interest:**

None Declared

